# The complete genome of a *Serratia marcescens* strain isolated from mosquito excreta

**DOI:** 10.1128/mra.00743-25

**Published:** 2025-09-23

**Authors:** Miranda M. Barnes, Mohamed Abdallah Mohamed Moustafa, Nicole E. Wagner, Dana C. Price

**Affiliations:** 1 Department of Entomology, Center for Vector Biology, Rutgers, The State University 242612, New Brunswick, New Jersey, USA; Indiana University, Bloomington, Bloomington, Indiana, USA

**Keywords:** *Serratia marcescens*, mosquito, vector biology, *Culex*, nonhuman microbiome

## Abstract

We report here the complete genome of a red-pigmented *Serratia marcescens* strain isolated from colony *Culex quinquefasciatus* mosquito excreta in New Jersey, U.S.A. The 4,947,975 bp circular chromosome encodes 4,711 genes. Homology searches suggest close relatedness to strain JW-CZ2, isolated from the soil rhizosphere in China.

## ANNOUNCEMENT

*Serratia marcescens* is a gram-negative bacterium isolated from diverse ecological contexts (1), including as an opportunistic pathogen in humans (2–4) and as a commensal or pathogen in insects (5, 6). In insects, research on *S. marcescens* has focused primarily on biopesticide development (7) and in the context of fighting vector-borne disease, as some strains have been associated with antimalarial activity (6, 8, 9). Given the interest in potentially leveraging *S. marcescens* for biocontrol and paratransgenesis, we report a complete genome sequence of *S. marcescens* isolated from colony-reared female *Culex quinquefasciatus* mosquito excreta.

Three female *Cx. quinquefasciatus* mosquitoes from a colony at the Rutgers University Center for Vector Biology were transferred singly to sterile plastic tubes with UV-irradiated bridal veil on both ends, provided with sterile 10% sterile sucrose solution via sterile dental cotton, and allowed to excrete while suspended over LB agar plates overnight in a dead air chamber. Two chambers + plates with no mosquitoes served as controls, and 200 µL of the sucrose solution was plated. After 16 hours, excreta droplets were visible on the agar surface, and plates were moved to a 34°C incubator. After 24 hours, red pigmented colonies had grown from the excreta on all mosquito plates; control plates remained negative. A single colony was serially passaged to create a clonal plate, and 50 colonies were isolated, pooled, and extracted using the Monarch Spin gDNA Extraction Kit (New England Biolabs, Ipswich, MA).

We generated 242,587 reads summing to 2.37 Gbp (read N50 = 16.29 Kbp) on an Oxford Nanopore MinION device using the SQK-LSK109 ligation kit with a FLO-MIN106 flow cell. The DNA was not sheared or size-selected prior to library preparation. Basecalling and adapter removal were performed using Guppy v6.0.1 (https://nanoporetech.com/software/other/guppy) in super accuracy mode. An Illumina DNA library was constructed using the DNA Sample Prep kit (Illumina Inc., San Diego, CA) and MiSeq sequencing generated ca. 407,364 paired-end reads (300 × 300 bp) summing to 81.8 Mbp (ca. 16× coverage of the below assembly). Reads were adapted and quality trimmed using bbduk (https://archive.jgi.doe.gov/data-and-tools/software-tools/bbtools/) and parameters (minlen = 50, qtrim = rl, trimq = 15, *k* = 20, and mink = 6). The uncorrected nanopore data were assembled with Canu v2.2 ([10]; default parameters; expected genome size = 5 Mbp) and polished with Illumina reads using Pilon v1.24 (11). The final assembly consisted of a single circular chromosome for which overlap was manually identified and trimmed, summing to 4,947,975 bp with GC content of 59.9%. Annotation was performed using the NCBI Prokaryotic Genome Annotation Pipeline v5.2 (12), identifying 4,711 genes (4,562 protein-coding, 22 rRNAs, 94 tRNAs, and 15 non-coding RNAs). The 16S small subunit rRNA locus was queried against the NCBI “nt” database; all top hits (*e*-value = 0.0; identity = 1,542/1,545 bp) were to *S. marcescens* species. The genome size of 4.95 Mbp makes this the second smallest fully assembled *S. marcescens* genome reported. The nucleotide sequence for each predicted gene was queried against the NCBI “nt” database using BLASTn ([13]; *e*-value = 1 × 10^−5^); the largest number of top-scoring hits (*n* = 1,795) was to *S. marcescens* strain JW-CZ2 (accession CP055161.1), isolated from soil rhizosphere and with enhancive effects on plant growth via antifungal properties and deaminase activity (14).

## Data Availability

The assembled genome, short- and long-read data generated from *S. marcescens* str. CVB in this work have been deposited to the NCBI under BioProject PRJNA1279212. The genome assembly has been assigned accession CP195229.
